# CytoGLMM: conditional differential analysis for flow and mass cytometry experiments

**DOI:** 10.1186/s12859-021-04067-x

**Published:** 2021-03-22

**Authors:** Christof Seiler, Anne-Maud Ferreira, Lisa M. Kronstad, Laura J. Simpson, Mathieu Le Gars, Elena Vendrame, Catherine A. Blish, Susan Holmes

**Affiliations:** 1 Department of Data Science and Knowledge Engineering, Maastricht University, Maastricht, The Netherlands; 2 Mathematics Centre Maastricht, Maastricht University, Maastricht, The Netherlands; 3 Department of Statistics, Stanford University, Stanford, USA; 4 Immunology Program, Stanford University School of Medicine, Stanford, USA; 5 Department of Medicine, Stanford University School of Medicine, Stanford, USA; 6 Chan Zuckerberg Biohub, San Francisco, USA; 7 Department of Microbiology and Immunology, Midwestern University, Downers Grove, USA

**Keywords:** High-dimensional cytometry, Generalized linear models, Generalized linear mixed models

## Abstract

**Background:**

Flow and mass cytometry are important modern immunology tools for measuring expression levels of multiple proteins on single cells. The goal is to better understand the mechanisms of responses on a single cell basis by studying differential expression of proteins. Most current data analysis tools compare expressions across many computationally discovered cell types. Our goal is to focus on just one cell type. Our narrower field of application allows us to define a more specific statistical model with easier to control statistical guarantees.

**Results:**

Differential analysis of marker expressions can be difficult due to marker correlations and inter-subject heterogeneity, particularly for studies of human immunology. We address these challenges with two multiple regression strategies: a bootstrapped generalized linear model and a generalized linear mixed model. On simulated datasets, we compare the robustness towards marker correlations and heterogeneity of both strategies. For paired experiments, we find that both strategies maintain the target false discovery rate under medium correlations and that mixed models are statistically more powerful under the correct model specification. For unpaired experiments, our results indicate that much larger patient sample sizes are required to detect differences. We illustrate the CytoGLMM *R* package and workflow for both strategies on a pregnancy dataset.

**Conclusion:**

Our approach to finding differential proteins in flow and mass cytometry data reduces biases arising from marker correlations and safeguards against false discoveries induced by patient heterogeneity.

## Background

Flow [1] and mass cytometry [2] allow researchers to simultaneously assess expression patterns of a large number of proteins on individual cells, allowing deep interrogation of cellular responses. The goal of such studies is to improve our understanding of the response mechanisms on a single cell basis by defining protein expression patterns that are associated with a particular stimulus or experimental condition. Finding differentially expressed proteins can help identify how cells function across experimental conditions. Some examples from our own work include: comparison between influenza strains [3], comparison between pregnant and non-pregnant women [4], comparison between healthy controls and HIV+ individuals [5], comparison between multiple sclerosis patients treated with daclizumab beta or placebo [6], and comparison between Beninese sex workers and healthy controls [7].

Statistical workflows that analyze data generated by flow and mass cytometry usually begin by clustering cells into both known and novel cell types. [8] provide an informative benchmark comparison study of many of the current clustering algorithms. The cluster step is followed by a differential expression analysis between and within cell types. The most popular differential analysis tools are: Citrus [9], the Bioconductor workflow by [10], cydar [11], CellCnn [12], and diffcyt [13].

We can classify differential analysis methods into marginal regression—analyses that focus on individual markers—and multiple regression—analyses that work on multiple markers simultaneously. The Bioconductor workflow by [10], cydar, and diffcyt are marginal regression methods. The advantage of marginal regression approaches is that they allow for flexible experimental designs—multiple factors, designs with interactions, designs with continuous variables, splines, and others are possible. The main disadvantage of this approach is in the separate testing for differential expression for each protein—when studying a specific protein marker—all the other markers are ignored. Therefore these methods are susceptible to biases induced by marker correlations.

Citrus and CellCnn are multiple regression methods. Their advantage is that they can provide a conditional interpretation of the effect of a protein onto the outcome, and thus reduce the bias due to marker correlations. A disadvantage is that Citrus summarizes protein expressions by taking the median for each cell type which can lead to a decrease in statistical power. The power decrease comes from the reduction in cell sample size from thousands of cells to one cell per sample. On the other hand, CellCnn uses a neural network for which it is currently unclear how to build confidence intervals, derive $p$-values, and control the number of falsely reported markers.

It is helpful to consider an example to further illustrate the differences between the marginal and the multiple regression method. Consider two intracellular proteins involved in interferon-$\gamma$ mediated signaling, *STAT1* and *IRF1*. Assume that applying a stimulus to *STAT1* activates transcription of *IRF1*. Further assume that the stimulus does not directly activate *IRF1*. If we performed separate differential analyses on protein *STAT1* and *IRF1*, we would observe differential expressions for both *STAT1* and *IRF1*, even though only *STAT1* had been directly activated. In contrast, a multiple regression method would report *STAT1* as differentially expressed given *IRF1*, and *IRF1* as not differentially expressed given *STAT1*.

CytoGLMM implements multiple regression that accounts for marker correlations without the aforementioned limitations. The main difference between our method and current methods is that we focus on cell-specific differential analysis and one fixed cell type, whereas current methods (Citrus, CellCnn, cydar, and diffcyt) learn cell types and perform differential analysis jointly. The narrower field of application allows us to define a more specific statistical model with easier to control statistical guarantees. Only the Bioconductor workflow by [10] focuses on specific cell types, but as mentioned before, they employ marginal regression which makes comparison to our multiple regression method difficult; as the two methods have different aims.

We present two versions of multiple regression: (1) A Generalized Linear Model (GLM) for unpaired samples. A GLM is a regression model that allows for a response and error terms that follow different distributions than the normal. (2) A restricted Generalized Linear Mixed Model (GLMM), which is a GLM that allows for random and fixed effects, for paired samples—when the same donor provides two samples, one for each condition. GLMs and GLMMs are generalizations of least squares to non-normal data. In our case, we will use logistic regression to model the experimental condition as unfair coin flips—when the coin flip comes up heads then the cell is declared to be stimulated, otherwise it is unstimulated. We model the coin fairness with a linear model of marker expressions after applying a transformation that ensures each coin flip has a probability of heads between zero and one.

Our models depart from the classic model where the marker expressions are the response variables. In our GLMs, the experimental condition is independent of the marker expression of interest given the other markers if the regression coefficient is zero (Proposition 2.2 in [14]). In contrast, the usual marginal regression analysis does not allow for such conditional statements. For instance, it would not allow us to rule out markers that are merely correlated with other makers but are independent of the experimental condition—as illustrated with the example earlier.

In summary, our two main contributions are:

1. We present a conditional differential analysis to avoid biases arising from marker correlations by using multiple regression instead of marginal regression.
2. We present two multiple regression strategies that work with the unsummarized expression data to maximize statistical power and account for patient heterogeneity to safeguard against false discoveries: (1) GLMs with a patient-level bootstrap, and (2) GLMMs with a patient-level random effect.

The “Results” section evaluates the statistical properties of both strategies implemented in our *R* package CytoGLMM on different simulated datasets, and illustrates the full workflow for real pregnancy data. In the “Discussion” section, we discuss our results in terms of biases and confounders. In the “Methods” section, we review the statistical background for GLMs and GLMMs.

## Results

We first evaluate the GLM and GLMM procedures for both paired and unpaired samples on simulated datasets. We then test them on a real pregnancy dataset.

### Simulated datasets

We generate simulated data with both cell and donor level variability. We allow for negative and positive correlations between markers and a wide range of correlation strengths. We simulate different scenarios ranging from weak to strong patient/cell variability. To make sure that we generate positive counts we use a Poisson noise model after transforming the generated expressions to positive real numbers using the exponential function. This is similar to using the log link function for Poisson GLMs. Overall, there are four main parameters: correlation $\rho _B$ and standard deviation $\sigma _B$ at the cell level, and correlation $\rho _U$ and standard deviation $\sigma _U$ at the donor level. Additionally, we can regulate the number of cells per sample and the number of donors per dataset. The differential expression signal is induced by shifting the mean vector on the logarithmic scale. We study the differential expression of three out of 10 markers after simulating exposure of cells to an experimental condition with two levels: stimulated versus unstimulated cells. The “Construction of simulated datasets” section provides a detailed mathematical description of the statistical model for the simulated datasets.

We perform simulations with a variety of different parameters. All simulations have 16 samples. For paired samples, those 16 samples come from 8 donors. For unpaired samples, those 16 samples come from 16 donors. Each sample has 1000 cells. We compared the observed False Discovery Rate (FDR) and the power. The FDR measures the statistical type 1 errors, the expected proportion of falsely declared discoveries over the total number of reported discoveries. The statistical power represents the proportion of correctly reported discoveries over the total number of true discoveries.

**Fig. 1:**
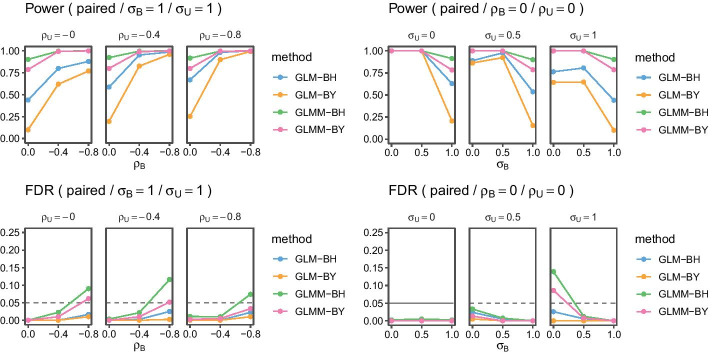
Summary of experiments with 1000 cells per sample averaged over 100 runs. The horizontal dashed line represents the target FDR. Postfixes BH and BY stand for the respective FDR control procedure. Subscripts *B* and *U* indicate cell and donor-level standard deviation $\sigma$ and correlation $\rho$, respectively

**Fig. 2:**
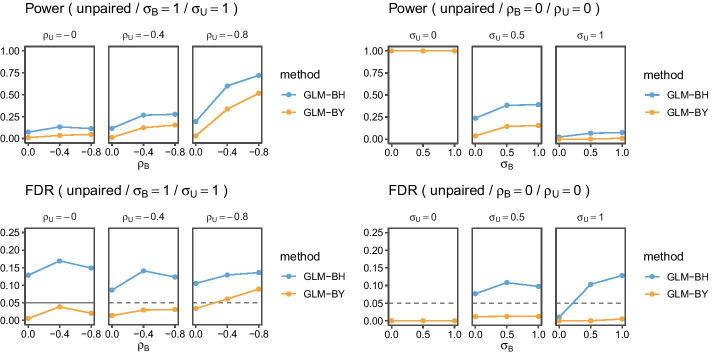
Summary of experiments with 1000 cells per sample averaged over 100 runs. The horizontal dashed line represents the target FDR. Postfixes BH and BY stand for the respective FDR control procedure. Subscripts *B* and *U* indicate cell and donor-level standard deviation $\sigma$ and correlation $\rho$, respectively

Figures 1 and 2 show a summary averaged over 100 runs for paired sample and unpaired sample experiments with effect size $\delta _p^{(1)}-\delta _p^{(0)} = 1.8$ and $\delta _p^{(1)}-\delta _p^{(0)} = 15$, respectively, and varying standard deviation $\sigma$ and correlation $\rho$ parameters. The dashed lines indicate the target FDR of $0.05$.

**Fig. 3:**
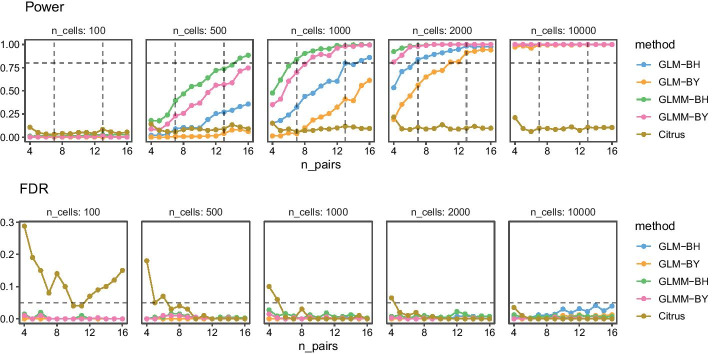
Summary of experiments with varying number of cells per sample averaged over 100 runs. Power: The horizontal dashed line represents a power of 0.8. FDR: The horizontal dashed line represents the target FDR of 0.05

First, let’s consider the paired samples experiment. The plots on the left show results when we vary cell and donor-level correlations at a fixed amount of cell $\sigma _B = 1$ and donor $\sigma _U = 1$ marker standard deviations. We observe only small differences across donor correlations $\rho _U$ of a small increase of power with increasing correlation. In contrast, there are large increases of power as a function of cell correlations $\rho _B$. In the panel of plots on the right, we set both correlations to zero and vary the marker standard deviations. In this setting, we again observe major changes with increasing standard deviations at the cell-level $\sigma _B$: the larger the cell-level variability, the lower the power. This is also true for donor-level variability, though to a much lesser extent. FDR is controlled below its target level under medium cell-level marker correlations ($| \rho _B | \le 0.4$) except when cell variability is at zero $\sigma _B = 0$, and donor variability is at one $\sigma _U = 1$. As expected, the Benjamini–Yekutiel (BY) procedure is more conservative than the Benjamini-Hochberg (BH) procedure, that is both FDR and power are lower. Interestingly, power increases with cell-level correlations $\rho _B$, and is virtually unaffected by donor-level correlations $\rho _U$. Overall, GLMM methods are more powerful than GLM methods. Figure 3 shows simulations for power and FDR with varying numbers of cells per samples and paired samples. Both cell and donor standard deviations are set to $\sigma _B = \sigma _U = 1$, and correlations are set to $\rho _B = \rho _U = 0$. We use the same effect size of $\delta _p^{(1)}-\delta _p^{(0)} = 1.8$ as in the experiment of Fig. 1. An efficiency gain is clearly visible when we compare how many paired samples are needed to achieve 80% power. We observe that with 1000 cells, GLMM-BH needs seven paired samples to exceed the 80% power threshold, whereas GLM-BH needs 13 paired samples to achieve the same. We can also see that GLMM-BH achieves adequate power with as few as 1000 cells per sample. We add results for Citrus to illustrate the power gain. Note that we chose the regularization parameter using leave-one-out cross-validation and select the parameter with the smallest prediction error. The original Citrus implementation chooses the regularization parameter using an FDR calculation. In our simulation study, the original procedure yields zero power across all sample sizes.

In the unpaired samples experiment, we only show GLM results as the GLMM results have zero power, there is no data to estimate the donor-level random effect term. We observe up to 20% FDR with a target FDR of 5%. To have non-zero power we need to increase the effect size to 15 (in comparison, for paired experiments the effect size is set to 1.8). Furthermore, FDR is only controlled under medium cell-level marker correlations using the more conservative BY procedure, with BH exceeding $0.05$ in most scenarios except when we have zero donor-level variability $\sigma _U = 0$. As before, BY comes with a loss of power.

### Experimental dataset

We reanalyze a published dataset on the maternal immune system during pregnancy [15]. The study provides a rich mass cytometry dataset collected at four time points during pregnancy in two cohorts. The authors isolated cells from blood samples and stimulated them with several activation factors. The goal was to explain how immune cells react to these stimuli, and how these reactions change throughout pregnancy. Findings from such experiments might identify immunological deviations implicated in pregnancy-related pathologies.

The data were collected at early, mid, late pregnancy, and six weeks postpartum. Samples were left unstimulated or stimulated. Stimulation conditions included: $\text {interferon-}\alpha \text {2A}$ ($\text {IFN}\alpha$), lipopolysaccharide, and a cocktail of interleukins (ILs) containing IL-2 and IL-6. They processed the samples on a CyTOF 2.0 mass cytometer instrument, and bead normalized the data to account for signal variation over time from changes in instrument performance [16].

In our analysis, we focus on comparing early (first trimester, $Y_i = 0$) with late (third trimester, $Y_i = 1$) pregnancy samples stimulated with IFN$\alpha$ in the first cohort of 16 women. We gate cells into cell types and organize them in a data frame. We follow the gating scheme detailed in [15] and define 12 cell types using the *R* package openCyto [17]: memory CD4 positive T cells (CD4+Tmem), naive CD4 positive T cells (CD4+Tnaive), memory CD8 positive T cells (CD8+Tmem), naive CD8 positive T cells (CD8+Tnaive), $\gamma \delta$T cells (gdT), regulatory T memory cells (Tregsmem), regulatory T naive cells (Tregsnaive), B cells, classical monocytes (cMC), intermediate monocytes (intMC), non-classical monocytes (ncMC), and Natural Killer cells (NK). Out of the 32 protein markers measured on each cell, the authors defined 22 markers as gating markers, and 10 as functional markers. The functional markers are pSTAT1, pSTAT3, pSTAT5, pNF$\kappa$B, total I$\kappa$B, pMAPKAPK2, pP38, prpS6, pERK1/2, and pCREB (in plots Greek symbols are replaced by Latin symbols).

**Fig. 4:**
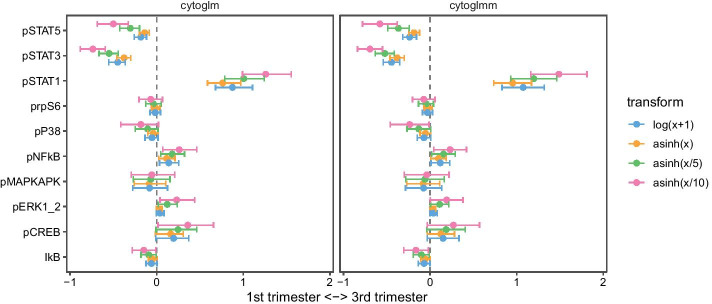
Methods comparison between bootstrap GLM (cytoglm) and GLMM (cytoglmm). The horizontal axes are on the log-odds scale. The vertical axes are the protein markers. Each color represents a different data transformation prior to the model fits

We plot the maximum likelihood (for GLMs) and the method of moments estimates (for GLMMs) with 95% confidence intervals for the fixed effects $\varvec{\beta }$ (Fig. 4). We transform the raw counts using four different transformations—a $\log$ transform and three asinh transforms with varying cofactor. The estimates are on the $\log$-odds scale. All four transformations show similar trends. The $\log$ transform is between the asinh with cofactor 1 and 5. We see that pSTAT1 is a strong predictor of the third trimester. With the standard cofactor of 5, this means that one unit increase in the transformed marker expression makes it between $\exp (1) = 2.7$ to $\exp (1.5) = 4.5$ (95% confidence interval for GLMM) more likely to be a cell from the third trimester, while holding the other markers constant. pSTAT3 and pSTAT5 have negative coefficients. This means pSTAT3 and pSTAT5 predict the first trimester, while holding the other markers constant. Only pSTAT1, pSTAT3, and pSTAT5 are below an FDR of 0.05. Our results corroborate previous findings by [15] reporting an increase of pSTAT1 during the third trimester for IFN$\alpha$ stimulated samples.

The GLMM method takes 1–2 s for the pregnancy dataset with 178,872 NK cells. The GLM requires resampling the data multiple times. For 1000 bootstrap replicates it takes 5 min for the pregnancy dataset. We obtained these running times on a laptop with an 2.3 GHz quad-core processor.

## Discussion

Our new *R* package CytoGLMM provides functions which are applicable to a wide range of cytometry studies. Besides comparisons on paired samples, where samples are available for the same subject under different experimental conditions, our CytoGLMM is applicable to unpaired samples, where samples are collected on two separate groups of individuals.

Our simulation experiments compare multiple regression GLM and GLMM, as implemented in cytoglm and cytoglmm in our *R* package. In simulated paired samples experiments, both GLMM with Benjamini-Hochberg (GLMM-BH) and Benjamini–Yekutieli (GLMM-BY) procedures control the FDR below the target FDR under cell-level marker correlations with an autoregressive structure with correlations up to $\pm 0.4$. GLMM methods are more powerful than GLM methods for paired samples. GLMM methods can account for the patient-to-patient variation in the model, whereas GLM methods treat this variation as noise, which results in noisier and thus less powerful estimates. For unpaired samples, we are forced to use the nonparametric bootstrap method for GLMs because there are no paired samples available to estimate the random effect term. In simulated unpaired experiments, only BY controls the target FDR. In practice, this means that we need a much higher donor samples size to detect a differential expression compared to paired experiments.

Interestingly, our power analysis suggests that GLMM-BH achieves adequate power with 1000 cells per sample. Not much can be gained by going to 10,000 or more cells. Such cell counts are not uncommon in cytometry studies. Our findings suggest that CytoGLMM will not detect any differential expression for rare cell types with around 100 cells per sample. Citrus showed low power in our simulation analysis. This makes sense as Citrus was not intended to be used for predefined cell types—its main focus is cell type discovery.

Overall, larger cell-level and donor-level correlations increase power and reduce the observed FDR. Hypothesis testing under arbitrary dependency structure is still an active research topic [14, 18, 19]. What is easier to explain is the reduction in power and FDR as a function of increased cell-level variance. Research in measurement error models shows that increased uncertainty in measured covariates is linked to biased estimates [20, 21]. For example, consider a scatter plot of experimental outcome (vertical axis) and one marker expression (horizontal axis). The goal is to fit a line so that we can predict the experimental outcome from the marker expression. Now assume that we measured the same marker with increased measurement error. This would spread out the the points along the horizontal axis, flatten the line fit, tilt it toward zero, and bias the regression coefficient towards zero. In GLMMs, donor-level correlations have only a weak impact on power and observed FDR because we explicitly model correlations with a random effect term.

In addition to corroborating a differential expression of pSTAT1 in the original study [15], we also found that pSTAT3 and pSTAT5 were differentially expressed in the NK cell population. This additional finding could be a result of the improved power of our method, but it could also be a result of the different regression analysis strategy. In the original study, the authors analyzed all cell types simultaneously. Such conditioning on other cell types could influence the differential expression estimates. In general, biases in coefficient estimates of GLMs and GLMMs can occur when we leave out proteins from the analysis. Assume that we would like to relate variable protein $X$ to experimental condition $Y$. If there exists a second protein $Z$ both related to $X$ and $Y$, then $Z$ is called a confounder, and not including it in the analysis can change the coefficients estimates. In the pregnancy data, if we removed pSTAT1 from the analysis, the confidence intervals of pSTAT3 and pSTAT5 could change. Such a difference is expected if pSTAT1 is a confounder. If pSTAT1 is not a confounder, the coefficient estimates for pSTAT3 and pSTAT5 will be the same whether pSTAT1 is included or not. The change of coefficients depending on what markers are included in the model can have strong effects. We observed in some real datasets that one marker can make other markers change their sign depending on whether we include them or not. In the pregnancy data, pSTAT5 flips sign from negative to positive after removing pSTAT1 from the analysis. In such cases, we recommend keeping all markers in the analysis to avoid introducing confounding biases.

We analyze 10 functional markers in the pregnancy data. CytoGLMM scales computationally to larger number of markers as GLMM can be implemented with the method of moments, and GLM with fast numerical optimization procedures. For example, a GLMM analysis on 40 markers, 16 samples, and 10,000 cells per sample takes 10 s on a laptop with an 2.3 GHz quad-core processor. There is however a statistical tradeoff as the effective sample size will be anywhere between the number of samples and the cells. To extend our methodology to more than two groups, we recommend to run a separate two-group CytoGLMM analysis on each pair, and combine the *p*-value tables—using the summary function—to control the overall FDR.

Our simulations are limited to a Poisson mixed effect model for protein marker expression. Our conclusions are only valid with respect to this model. The real data generating process might be different. Two main caveats are to be noted. First, we can only encode an experimental design comparing two groups. Second, we require gated cell types. To reduce the person-to-person bias of manual gating, we employed the *R* package openCyto [17]. The curse of dimensionality makes it challenging to scale this approach to very high dimensional gating schemes. For example, consider 20 gating markers and assume that each marker differentiates between two cell populations. This seemingly harmless gating procedure can produce $2^{20}$ or approximately one million possible cell types. In this setting, even large cell sample sizes might provide unreliable cell types estimates.

A possible alternative to GLMMs are Generalized Estimating Equations (GEEs). GEEs are statistically more efficient when the covariance structure of the residuals are known. In our case, the covariance structure is unknown and needs to be estimated from the data. In most immunology studies, we only have a few donors without a given covariance structure (e.g. no time dependency), resulting in a hard and possibly unstable covariance estimation problem, which could result in an overall loss of efficiency [22].

## Conclusions

We presented a conditional differential analysis to avoid biases arising from marker correlations. We built statistical models of the unsummarized expression data to maximize statistical power, and modeled patient heterogeneity to safeguard against false discoveries. The main difference between our and related procedures is that we assume that the cell type is known or can be estimated with high accuracy. This assumption is reasonable in many studies with cytometry data. In our own work, we applied CytoGLMM in wide range of immunology studies: In [3], we identified differential expressions in CD112 and CD54 between the pandemic A/California/07/2009 and the seasonal A/Victoria/361/2011 influenza virus strains. In [4], we found increased expression of CD38 on CD56dim and CD56bright NK cells, and NKp46 on CD56dim NK cells in pregnant women compared to non-pregnant women. In [5], we found that TIGIT is upregulated on NK cells of untreated HIV+ women, but not in antiretroviral-treated women. In [6], we found that treatment with daclizumab beta increased expression of NKG2A and NKp44, and diminished expression of CD244, CD57, and NKp46 on CD56bright NK cells. Most recently, in [7], we found that in a cohort of Beninese sex workers and healthy controls NK cells from highly exposed seronegative individuals had increased expression of NKG2A, NKp30 and LILRB1, as well as the Fc receptor CD16, and decreased expression of DNAM-1, CD94, Siglec-7, and NKp44.

Both the GLM and GLMM method build on generalized linear models that can model other data types than binary responses. Therefore it would be possible to extend CytoGLMM to continuous response variables. A more challenging next step is extending CytoGLMM to include more complicated experimental designs; e.g. twin studies [23].

## Methods

### Preprocessing

We recommend that marker expressions be corrected for batch effects [10, 24–27] and transformed using variance stabilizing transformations to account for heteroskedasticity, for instance with an inverse hyperbolic sine transformation with the cofactor set to 150 for flow cytometry, and 5 for mass cytometry [2]. This transformation assumes a two-component model for the measurement error [28, 29] where small counts are less noisy than large counts. Intuitively, this corresponds to a noise model with additive and multiplicative noise depending on the magnitude of the marker expression; see [30] for details.

### Generalized linear model (GLM)

The goal of the GLM is to find protein expression patterns that are associated with the condition of interest, such as a response to a stimulus. We set up the GLM to predict the experimental condition from protein marker expressions, thus our experimental conditions are response variables and marker expressions are explanatory variables. The response variable $Y_i$ is a binary variable encoding experimental condition as zero or one. The response variable can be modeled as a Bernoulli random variable with probability $\pi _i$ for each cell. Then we use the ${\text {logit}}$ link to relate the linear model to binary responses. The linear model predicts the logarithm of the odds of the $i$th cell being $Y_i = 1$ instead of $Y_i = 0$. The linear model has one coefficient per protein marker $\beta _1,\dots ,\beta _P$ and an intercept $\beta _0$. If $\pi _i$ is 0.5 then the cell could have come from either $Y_i = 1$ or $Y_i = 0$ with equal probability. If $\pi _i$ is either very close to one or zero, then the cell is strongly representative of a cell observed under $Y_i = 1$ or $Y_i = 0$, respectively. We observe the protein marker expressions ${\varvec{x}}_i$. For each cell we measure $P$ protein markers.

The response probabilities $\pi _i$ are not observed directly, only $Y_i = y_i$ and ${\varvec{x}}_i$ are observed. Note that ${\varvec{x}}_i$ is observed with errors. Here, we make the approximating assumption that the covariates are fixed. Our results will show that this assumption is conservative and introduces a regularization of the estimated coefficients. We estimate $\pi _i$ from the data using maximum likelihood with the function glm in *R*. Our logistic regression model, which is part of a general class of GLMs, can be summarized in the following form:

$$\begin{aligned} \begin{aligned} Y_{i}&\sim {\text {Bernoulli}}(\pi _{i}), \\ \log \left( \frac{\pi _i}{1-\pi _i}\right)&= {\varvec{x}}_i^T\varvec{\beta }. \end{aligned} \end{aligned}$$

For likelihood inference, we use the nonparametric bootstrap and resample entire donors with replacement to preserve the cluster structure. At the cell-level, we resample cells with replacement within each donor. We build percentile confidence intervals and compute $p$-values by inverting the intervals assuming two-sided intervals with equal tails [31]. We use the BH [32] and BY [33] procedures to control the FDR. We refer to GLM with BH control as GLM-BH, and with BY control as GLM-BY.

### Generalized linear mixed model (GLMM)

We make additional modeling assumptions by adding a random effect term in the standard logistic regression model to account for the subject effect. The covariates ${\varvec{x}}_{ij}$ are the same as in the fixed effects GLM, except now we have an additional index $j$ that indicates from which donor the cell was taken. Each cell $i$ maps to a donor $j$. The additional term ${\varvec{u}}_j$ represents regression coefficients that vary by donor. The statistical model can be summarized as,

$$\begin{aligned} \begin{aligned} Y_{ij}&\sim {\text {Bernoulli}}(\pi _{ij}), \\ \log \left( \frac{\pi _{ij}}{1-\pi _{ij}}\right)&= x_{ij}^T\varvec{\beta } + x_{ij}^T{\varvec{u}}_{j}, \end{aligned} \end{aligned}$$

with a multivariate normal distribution and covariance matrix $\varvec{\Sigma }$ for the random effect term ${\varvec{u}}_j$,

$$\begin{aligned} {\varvec{u}}_j \, | \, \varvec{\Sigma } \sim {\text {Normal}}\left( {\varvec{0}}, \varvec{\Sigma } \right) . \end{aligned}$$

Analog to our GLM, we make the approximating assumption that the covariates are fixed.

The mixed effect model is a compromise between two extremes. On the one hand, we could estimate separate regression coefficients for each donor. This corresponds to random effects modeled with a multivariate normal distribution with infinite standard deviations and no constraint on how coefficients are related to each other. On the other hand, we could pool all donors into one group and ignore the donor information. This corresponds to a GLM with no random effects, with no additional variability besides the fixed effect term. A compromise between these two extremes is to estimate the standard deviations of the random effects from data, allowing the regression model to learn from the other donors. Mixed effects procedures are related to empirical Bayes procedures [13]. The first step of an empirical Bayes procedure would estimate the mean and covariance matrix of the random effect term. The second step would fix the random effect parameters at their estimated values and estimate the fixed effect parameters. In contrast, the mixed effect procedure estimates the parameters of both steps jointly. This is possible for flow and mass cytometry data because of the relatively small number of proteins.

We use the method of moments as implemented in the *R* package mbest to estimate the model parameters $\varvec{\beta }$, ${\varvec{u}}_j$, and $\varvec{\Sigma }$. For likelihood inference, we use the asymptotic theory derived by [34]. The author showed that the sampling distribution of the estimated parameters can be approximated by a normal distribution. We use this mathematical alternative to the bootstrap method to create approximate confidence intervals and $p$-values. As in the GLM case, we use the BH and BY procedures to control the FDR. We refer to GLMM with BH control as GLMM-BH, and with BY control as GLMM-BY.

### Construction of simulated datasets

**Fig. 5:**
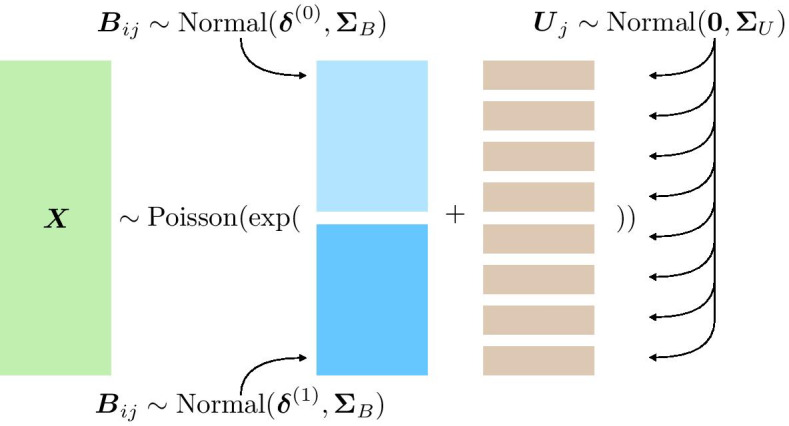
Graphical representation of the Poisson GLM used to construct the simulated datasets

We construct our simulated datasets by sampling from Poisson GLMs. In prior work, we confirmed—with predictive posterior checks—that Poisson GLMs with mixed effects provide a good fit to mass cytometry data on the same pregnancy dataset [35]. We consider one underlying data generating mechanisms described by a hierarchical model for the $i$th cell and $j$th donor:

$$\begin{aligned} \begin{aligned} {\varvec{X}}_{ij}&\sim \text {Poisson}(\varvec{\lambda }_{ij}) \\ \log (\varvec{\lambda }_{ij})&= {\varvec{B}}_{ij} + {\varvec{U}}_j \\ {\varvec{B}}_{ij}&\sim {\left\{ \begin{array}{ll} \text {Normal}(\varvec{\delta }^{(0)}, \varvec{\Sigma }_B) &{} \text {if } Y_{ij} = 0, \text { cell unstimulated} \\ \text {Normal}(\varvec{\delta }^{(1)}, \varvec{\Sigma }_B) &{} \text {if } Y_{ij} = 1, \text { cell stimulated} \end{array}\right. } \\ {\varvec{U}}_j&\sim \text {Normal}({\varvec{0}}, \varvec{\Sigma }_U). \end{aligned} \end{aligned}$$

Figure 5 shows a graphical representation of the hierarchical model. The stimulus activates proteins and induces a difference in marker expression. We define the effect size to be the difference between expected expression levels of stimulated versus unstimulated cells on the $\log$-scale. All markers that belong to the active set *C*, have a non-zero effect size, whereas, all markers that are not, have a zero effect size:

$$\begin{aligned} {\left\{ \begin{array}{ll} \delta ^{(1)}_p - \delta ^{(0)}_p > 0 &{} \text {if protein } p \text { is in activation set } p \in C \\ \delta ^{(1)}_{p'} - \delta ^{(0)}_{p'} = 0 &{} \text {if protein } p' \text { is not in activation set } p' \notin C. \end{array}\right. } \end{aligned}$$

Both covariance matrices have an autoregressive structure,

$$\begin{aligned} \begin{aligned} \Omega _{rs}&= \rho ^{|r-s|} \\ \varvec{\Sigma }&= {\text {diag}}(\varvec{\sigma }) \, \varvec{\Omega } \, {\text {diag}}(\varvec{\sigma }), \end{aligned} \end{aligned}$$

where $\Omega _{rs}$ is the *r*th row and *s*th column of the correlation matrix $\varvec{\Omega }$. We regulate two separate correlation parameters: a cell-level $\rho _B$ and a donor-level $\rho _U$ coefficient. Non-zero $\rho _B$ or $\rho _U$ induce a correlation between condition and marker expression even for markers with a zero effect size.

### Processing of pregnancy dataset

We reproduce the original gating strategy according to the supplementary material (Figure S1) from [15] using the *R* package openCyto [17]. In our analysis, we focus on the 178,872 NK cells. The number of cells per sample vary between 6480 and 21,348. The full openCyto workflow is available as a vignette on our package website: https://christofseiler.github.io/CytoGLMM/articles/pregnancy_dataset.html.

## Data Availability

All data analysed during this study are included in [15]. All results and figures can be reproduced by running the manuscript *Rmd* available on GitHub: https://github.com/christofseiler/CytoGLMM_BMC/; Our *R* package is available on GitHub: https://github.com/christofseiler/CytoGLMM/; A vignette is available on our *R* package website: https://christofseiler.github.io/CytoGLMM/articles/CytoGLMM.html.

## Abbreviations

GLM: Generalized linear model
GLMM: Generalized linear mixed model
FDR: False discovery rate
BH: Benjamini-Hochberg
BY: Benjamini–Yekutieli
